# Assessing the Effectiveness of Flipped Classroom Among Undergraduate Medical Students: A Crossover Quasi-experimental Study

**DOI:** 10.7759/cureus.106691

**Published:** 2026-04-08

**Authors:** Rekha Dutt, Kaushik Mukhopadhyay, Babaji Ghewade, Vaibhav P Anjankar, Adarshlata Singh, Chandrika Das, Semanti Das

**Affiliations:** 1 Community and Family Medicine, All India Institute of Medical Sciences, Kalyani, Kalyani, IND; 2 Pharmacology, All India Institute of Medical Sciences, Kalyani, Kalyani, IND; 3 Respiratory Medicine, Jawaharlal Nehru Medical College, Datta Meghe Institute of Higher Education and Research, Wardha, IND; 4 Anatomy, Jawaharlal Nehru Medical College, Datta Meghe Institute of Higher Education and Research, Wardha, IND; 5 Dermatology, Venereology and Leprosy, Jawaharlal Nehru Medical College, Datta Meghe Institute of Higher Education and Research, Wardha, IND; 6 Community Medicine, All India Institute of Medical Sciences, New Delhi, New Delhi, IND

**Keywords:** community medicine, didactic, flipped classroom, higher order cognitive skills, lower order cognitive skills

## Abstract

Background: Flipped classroom (FC) is the pedagogical shift of passive content to the pre-class phase and active learning during the class. During the pre-class session, students develop understanding, i.e., lower-order cognitive skills (LOCS). In class time, they are expected to develop application, analysis, evaluation*, *i.e., the higher-order cognitive skills (HOCS). The objectives were to compare HOCS gained through flipped classroom and didactic lectures (DL) and to explore learners’ perceptions of FC.

Method: This study was conducted in the Community Medicine and Family Medicine (CMFM) department of a medical college in West Bengal, India. A cross-over quasi-experimental design was used with fourth-semester medical students in CMFM practical classes. A total of 125 students were divided into FC and DL groups with crossover after a fortnight during the second session. Pre- and post-tests with 10 validated multiple-choice questions were conducted, and student feedback was collected on a four-point Likert scale.

Results: In the first session, the mean difference in scores in the interventional and control groups was 1.43 (SD 2.22) and 1.37 (SD 2.14), respectively, with a p-value of 0.53526. In the crossover second session, the mean difference in scores in the interventional and control groups was 0.94 (SD 1.66) and 1.18 (SD 2.01), respectively, with a p-value of 0.9442.

Conclusions: Student participation in FC was low, and there was no conclusive difference in score improvements between FC and DL.

## Introduction

There is a shift from traditional curriculum to competency-based medical education, which focuses on developing entrustable professional activities (EPA), fostering the habit of self-directed learning [1]. Self-directed learning, self-monitoring, development of metacognitive skills, and reflective practices are the main components of lifelong learning, and all of these can be inculcated in the learners by using appropriate teaching-learning methods [2]. 

Bloom’s revised taxonomy theory has six levels of learning, from the lowest to the highest level in the form of a pyramid, i.e., Remember, Understand, Apply, Analyze, Evaluate, and Create [3]. Through these levels, the learner recalls the information, understands the concepts, applies knowledge to real situations, analyzes various components through critical thinking, and produces new theory or product. According to Bloom’s revised taxonomy, to remember and to understand are considered lower-order cognitive skills (LOCS), while to apply (problem-solving), to analyze (critical thinking), to evaluate, and to create are considered higher-order cognitive skills (HOCS) [4].

A flipped classroom (FC) or Inverted classroom is an instructional technique that develops critical thinking skills among students by engaging them in an active learning process. FC has two components: pre-class learning and in-class learning [5]. In FC, reading and understanding the information is done before the class, whereas the higher levels of learning, like application, analysis, and evaluation of the information, are carried out during the class time [1].

As part of the undergraduate medical curriculum, biostatistics is taught in the Department of Community Medicine and Family Medicine (CMFM). Biostatistics is a concept-based topic in which basic knowledge is applied for problem-solving in new scenarios and acquiring analytic skills to interpret the data. The present study was conducted in the CMFM department with the following objectives: (i) To compare the change in knowledge of undergraduate medical students with the FC model and traditional DL model by assessing the scores in higher-order cognitive skills (application and analysis) in biostatistics, and (ii) To collate the perception of undergraduate medical students and instructor on the introduction of FC model in the CMFM department.

The results of this study were presented in a free faculty paper oral presentation at the 39th West Bengal Chapter conference of the Indian Association of Preventive and Social Medicine, in Kolkata on August 10, 2024.

## Materials and methods

This was a two-arm quasi-experimental study, crossover design, conducted in the CMFM department at All India Institute of Medical Sciences, Kalyani, West Bengal, India, that included the Second Professional (fourth semester) undergraduate medical students. The study was approved by the Ethics Committee of All India Institute of Medical Sciences, Kalyani, West Bengal (registration number: IEC/AIIMS/Kalyani/certificate/2024/299).

Sample size and division

Based on the roll numbers, a total of 125 students were divided into two groups: “Group A” and “Group B,” comprising roll numbers 1-63 and 63-125, respectively. Both groups had scheduled practical classes of three hours once a week on different days. The objectives and details of the study were explained to participants, and informed consent was taken before the commencement of the study. It was voluntary participation in the project. It was informed that scores obtained by students would be used only for research and would not affect their internal assessment. (NOTE: There are three Professionals divided into semesters for the undergraduate medical course in India. The First Professional has semesters 1 and 2. The Second Professional has semesters 3 and 4. The Third Professional spans semesters 5-9. Community Medicine is taught from semester 1 to 7. We conducted this study in semester 4 (Second Professional).)

Study implementation

In the first phase, Group A was taught topic 1 (Data Presentation) in the FC model and Group B was taught the same in DL model. In the second phase, Group B was taught topic 2 (Sampling techniques) in the FC model and Group A was taught the same in DL model. Different topics were taken in crossover to align with the pre-approved class schedule. The study implementation is summarized in Table 1.

**Table 1:** Chronological implementation of flipped classroom and lecture-based model in the study MCQ: multiple-choice questions; HOCS: higher-order cognitive skills; LOCS: lower-order cognitive skills

Crossover in the next class	Flipped Classroom (Intervention)	Lecture-based Model (Control)
Batch	Group A (n=62)	Group B (n=63)
One week before the session	The introduction and objectives of the study were explained. Informed consent taken. Separate WhatsApp groups created.	
Pre-test: A link with a 20-minute timer shared on WhatsApp	Ten pre-validated MCQs based on HOCS	
Activities before the scheduled class (6 days)	Sharing of Specific Learning Objectives. Link to a pre-recorded videos of 20 minutes plus a PDF of the PowerPoint presentation. Read the topic from the standard textbook	Sharing of Specific Learning Objectives
Activities during the scheduled class (Two Hours)	Link to five MCQs based on LOCS	
	Teams of 5-6 students were made. Each team was given a set of topic-based scenarios (45 minutes). There was a presentation by each team (10 minutes), This was followed by replying to queries by the instructor and feedback on a 5-point Likert scale	The same instructor took the didactic class using the same PowerPoint presentation and case scenarios. Answering of queries by the instructor
Post Test: A link with a 20-minute timer shared on WhatsApp	Ten pre-validated MCQs based on HOCS	
After one week	Feedback from instructor, Open-ended feedback from students	

Pre-Test

Two separate WhatsApp groups were created for both groups of participants. The pre-test consisted of 10 MCQs to assess the students’ HOCS [6]. The link to the pre-test was shared on both WhatsApp groups (Meta Platforms, Inc., Menlo Park, California, United States) with a timer of 20 minutes.

FC Model

In the FC sessions, two videos based on specific learning objectives of the topic were recorded online by the instructor using the Zoom platform (Zoom Communications, Inc., San Jose, California, United States). PowerPoint presentations and Microsoft Excel sheets were used in the videos (Microsoft Corporation, Redmond, Washington, United States). Each video was 20 minutes in duration. The duration of the videos was kept short to keep the interest and compliance of students [7]. The videos and PowerPoint presentation were shared with the students in their respective WhatsApp groups five days before the class. The participants were instructed to watch the videos and read the topic from the standard textbooks.

Based on the Blooming Biology Tool (BBT), 10 multiple choice questions (MCQs) that required prediction of the most likely outcome given a new situation and interpretation of data with selection of the best conclusion were collated to test the higher levels of the cognitive domain, viz., to apply and to analyze [8]. Two external experts validated the specific learning objectives, content, and assessment items.

During the in-class session, the instructor assessed the LOCS gained by five MCQs based on the “understand” level after watching the video. Then, the students were divided into eight teams of five to six members, with the roles of a leader, a scribe, and a timekeeper. Each team solved the separate scenario-based questions during an assigned period of 45 minutes and presented them in front of the whole batch. The instructor discussed the queries. At the end of the session, the instructor summarized the responses from all the groups, answered queries, and provided feedback.

DL Model

In the lecture-based didactic classroom, the students attended a one-hour lecture followed by 15 minutes of answering queries by the instructor. The instructor who made the videos gave direct information through the lectures as well. This ensured uniformity in the course content and delivery of the instructions.

Post-Session Process

At the end of the sessions, the students were sent a link to the post-test, which had 10 MCQs with a timer of 20 minutes to assess HOCS. Crossover with the next topic was done after a fortnight in alignment with the pre-approved scheduled class by following the same steps.

The scores of students were analyzed. The normality of data distribution was evaluated using the Shapiro-Wilk test. As the results indicated a non-normal distribution, the non-parametric Mann-Whitney U test was employed for the statistical analysis of the scores.

The students were asked to fill out an online feedback form after the class to investigate the students’ attitudes towards the FC. The form contained statements regarding the learners’ experience, perceived benefit of the FC, course materials, teaching process, and evaluation system. The form used a five-point Likert scale (1=strongly disagree and 5=strongly agree). The internal consistency of the feedback form was calculated as Cronbach’s alpha=0.912. Open-ended feedback from students and the teacher was taken about their perception of FC.

## Results

Out of 125 students in the class, 118 provided informed consent to participate in the study. However, the number of students who completed both pre- and post-tests in Group A and in Group B was 47 and 64, respectively. Each student was supposed to appear twice, once as a participant in the intervention group and the second time in the crossover as a control. Table 2 shows the participation of students at different levels of the project. A total of 111 paired observations were analyzed with an attrition rate of 47%.

**Table 2:** The number of students who participated at various levels of the study

Session	Number of participants	Attempted both pre and post test	Scored >60% in LOCS. Percentage in parenthesis	Attempted only pretest	Attempted only posttest
Flipped classroom “Data Presentation”	52	30	29 (97)	15	7
Didactic lecture “Data Presentation”	63	46	18 (39)	10	7
Flipped classroom “Sampling Technique”	45	18	13 (72)	17	10
Didactic lecture “Sampling Technique	50	17	15 (88)	28	5
Total	210	111 (Included)		99 (Excluded)	

On the topic of “Data Presentation,” FC and DL were attempted by 30 and 17 participants, respectively. The mean pre- and post-test scores in FC were 4.9 (SD: 1.56) and 6.33 (SD: 2.36), respectively. The mean pre- and post-test scores in DL were 5.13 (SD: 1.63) and 6.5 (SD: 1.83), respectively. Minimum scores in the pre-test and post-test in FC were 2 and 0, whereas in DL, they were 1 and 3, respectively. Pre- and post-test scores in both groups had the same 25th percentile of 4 and 5, median 5 and 7, and 75th percentile of 6 and 8. The maximum scores attained in FC and DL in the pretest were 7 and 8, respectively, and post-test scores in both groups were 10 (Table 3).

**Table 3:** Scores of pre- and post-test in flipped classroom and didactic lecture models in the topic of “Data Presentation”

Group	Intervention Group (n=30)		Control Group (n=46)	
Scores	Pre-test	Post-test	Pre-test	Post-test
Mean (SD)	4.9 (1.56)	6.33 (2.26)	5.13 (1.63)	6.5 (1.83)
Minimum	2	0	1	3
25th Percentile (Q1)	4	5	4	5
Median (50th Percentile, Q2)	5	7	5	7
75th Percentile (Q3)	6	8	6	8
Maximum	7	10	8	10

**Figure 1:**
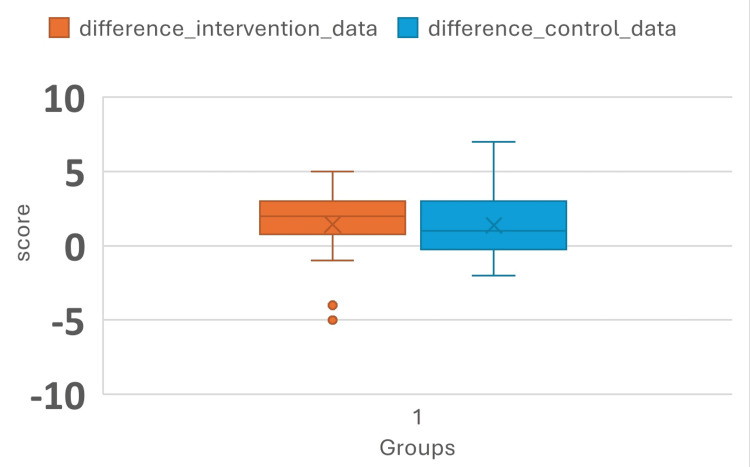
The difference in pre- and post-test scores in the flipped classroom and didactic models on the topic of “Data Presentation”

Figure 1 shows the difference in the scores of pre- and post-test in the FC and DL on the topic of Data Presentation. The mean difference in scores in the interventional and control groups was 1.43 (SD 2.22) and 1.37 (SD 2.14), respectively. The score difference in FC and DL at the 25th percentile was -5 and -2, the median was 2 and 1, the 75th percentile was 3 in both groups, whereas the maximum score difference was 5 and 7, respectively. In the Mann-Whitney U test, the test statistic was approximately 631, and the p-value was 0.53526.

On the topic of “Sampling Techniques,” FC and DL were attempted by 18 and 17 participants, respectively. Table 4 shows that the mean pre- and post-test scores in FC were 6 (SD: 7.17) and 5.94 (SD: 7. 12), respectively. The SD for several data points was notably larger than the mean. This high variance relative to the mean indicates that the data was significantly skewed. In DL, the mean pre- and post-test scores were 5.94 (SD: 1.95) and 7.12 (SD: 1.83), respectively. The minimum pre- and post-test scores in FC were 4, whereas in DL, they were 3 and 4, respectively. Pre- and post-test scores at the 25th percentile in FC were 5 and 5.75, and in DL, they were 4 and 6; median of FC was 5.5 and 7.5, and that of DL was 6 and 7, 75th percentile in FC was 7.25 and 9, and DL were 8 and 8.5, the maximum scores attained in FC were 9 and 10 and DL were 9 and 10, respectively. 

**Table 4:** Pre- and post-test scores in flipped classroom and didactic lecture models in the topic of “Sampling Technique”

Group	Intervention (n=18)		Control (n=17)	
Scores	Pre-test	Post-test	Pre-test	Post-test
Mean (SD)	6 (1.64)	7.17 (1.82)	5.94 (1.95)	7.12 (1.83)
Minimum	4	4	3	4
25th Percentile (Q1)	5	5.75	4	6
Median (50th Percentile, Q2)	5.5	7.5	6	7
75th Percentile (Q3)	7.25	9	8	8.5
Maximum	9	10	9	10

**Figure 2:**
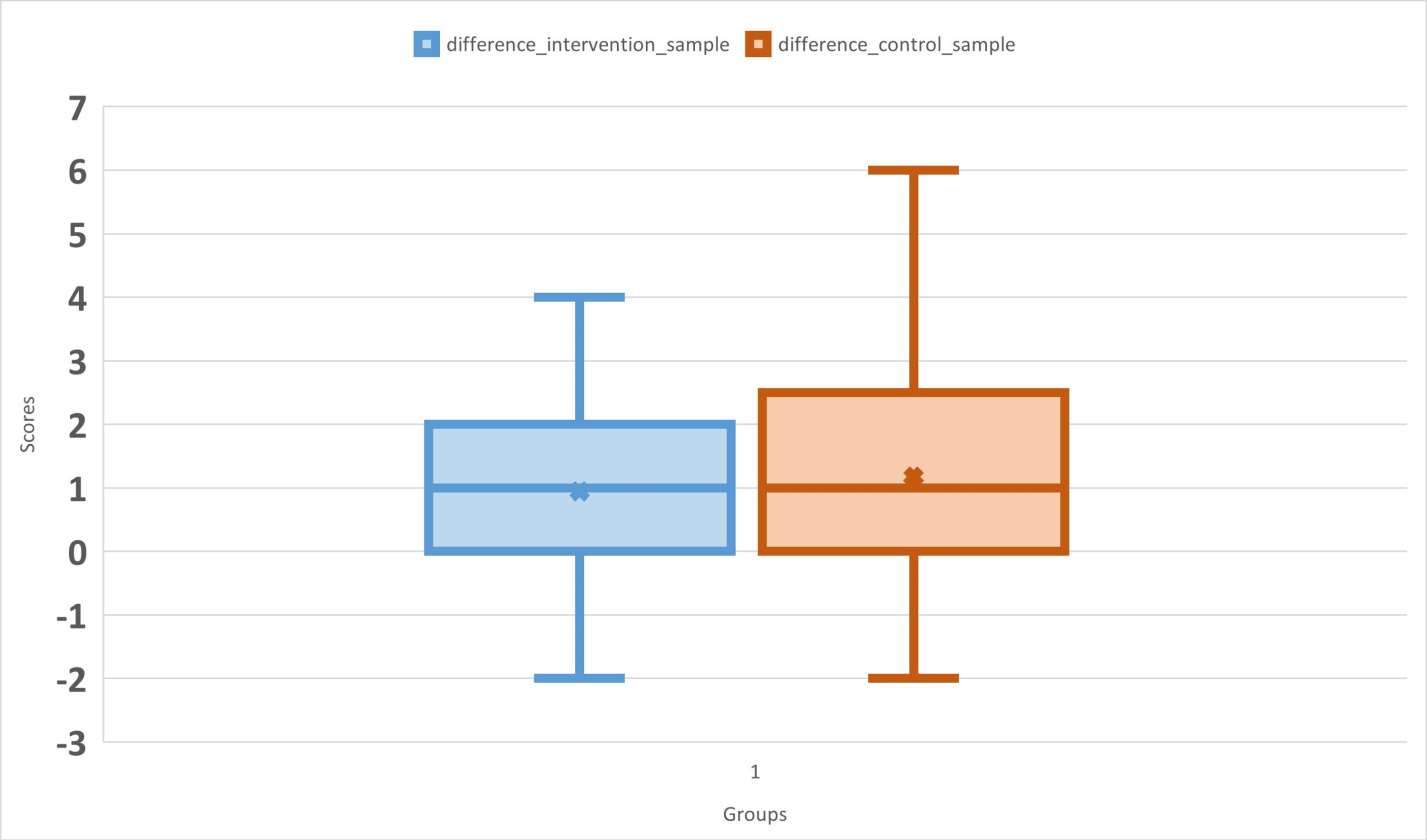
Difference in pre- and post-test scores in the flipped classroom and didactic lecture models in the topic of the “Sampling Technique”

Figure 2 shows the difference in the scores of pre- and post-tests in the FC and DL on the Sampling Technique. The mean difference in scores in the FC and DL models was 0.94 (SD 1.66) and 1.18 (SD 2.01), respectively. The score difference in the FC and DL models at a minimum level, 25th percentile, and the median was the same, i.e., -2, 0, and 1 respectively. The score difference at the 75th percentile was 2 and 2.5, whereas the maximum score difference was 4 and 6 in the FC and DL groups, respectively. In the Mann-Whitney U test, the test statistic was approximately 150.5, and the p-value was approximately 0.9442.

**Figure 3:**
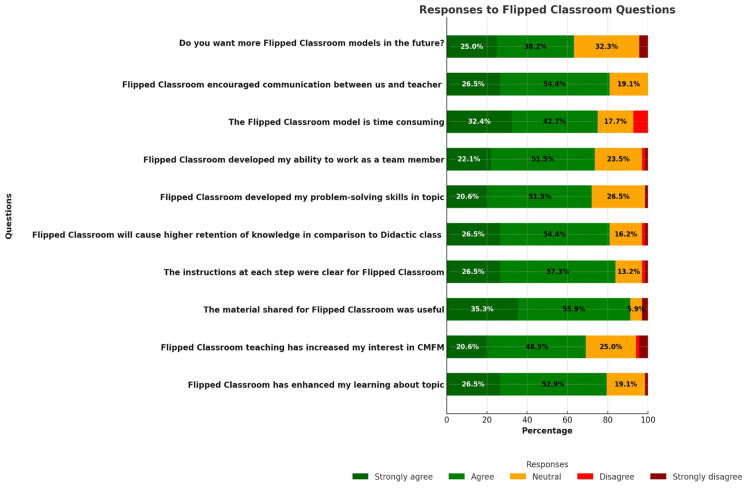
Feedback from students on a five-point Likert Scale (n=68)

Figure 3 shows the response to online feedback on the five-point Likert scale, which 68 students attempted. Most students agreed in favor of FC. However, 22 (32.3%) students were neutral and three (4.5%) strongly disagreed about having FC in the future. The Cronbach’s alpha for the given dataset is 0.912.

Online open-ended feedback shows that most students felt that FC fosters repetition and, hence, reinforcement of knowledge for problem solving, thus making the topic interesting. It provides resources for future reference and fosters learning at one's own pace. Limitations are that if any student misses the homework, then they would not understand the subject at all. The students would need both video and text as resources for understanding and making annotations for future reference.

Feedback responses on the reasons for low participation in the project indicated that most students were not interested in CMFM, as they prioritized their studies for the examination of the Second Professional subjects. A few students could not open the MCQs link due to the timer and weak internet. Most students opined that FC should be conducted during the Third Professional when the CMFM subject has a summative assessment. Students expected "double attendance" as an incentive for FC.

Open-ended feedback was taken from the teacher about her experience and perception of FC in CMFM. According to her experience, FC can only be fruitful if all students are motivated to participate. It can be successful in small-group teaching or exam-going batches with easier topics.

## Discussion

The FC promotes integrating independent self-paced learning, using technology before the class, and learner-centred activities during the class. In our study, 99 out of 210 (47%) students didn’t participate at all levels of the study due to voluntary participation in the project and weak internet.

Change in knowledge

In the first phase of the study, higher scores in LOCS were gained in the FC batch compared to DL. This shows that students followed the resources shared in FC. The score difference in the post-test was higher among FC than DL. However, in the second phase, only a few students participated, and students of the DL group obtained higher scores in LOCS. The difference in the post-test scores was higher in the DL group in comparison to the FC group, and the DL group gained higher scores in HOCS. However, in both phases, the results were not statistically significant.

The effects of the FC were mixed and inconclusive. In our study, 47% of students did not attempt both pre- and post-tests, so their scores were not included in the analysis. Student compliance with FC affects differential gains in knowledge compared to DL. In a study by Heitz et al., 31% of students did not attempt the tests, and the results on FC’s benefits were inconclusive [9]. Another study by Evans et al. also taught clinical epidemiology and biostatistics in blended learning and DL [10]. The results did not show any significant change in performance in both techniques, and the students preferred class lectures. Many studies concur with our findings of non-significant differences in the results of the pre- and post-tests in the FC and DL groups and long-term retention of knowledge [5,11-13].

Perception of students

Most students who participated in the study preferred to have FC in the future. Most of them agreed that FC enhanced their skills in problem-solving and encouraged communication with the teacher. FC also enhanced its interest in community medicine. The retention of knowledge was better with FC than with DL. Similarly, a systematic review and meta-analysis reported that multiple studies had shown the positive students’ perceptions of and attitudes toward the FC. Students especially found benefits in online modules as a learning resource [14]. 

A study reported a higher satisfaction rate among students but poor performance in blended learning compared to DL [9]. Similarly, a randomized controlled trial on teaching of evidence-based medicine through blended learning and DL reported greater perceived self-efficacy and application of the subject, but blended learning was not more effective than DL in increasing the knowledge and skills in the subject [15]. A scoping review concurs with our findings that positive students’ perception toward FC does not necessarily mean that this teaching-learning method would significantly improve their learning [16].

Reasons for low participation in the FC

In India, community medicine is taught from the First Professional to the Third Professional, and a summative examination is conducted at the end of semester 7 in the Third Professional. This study was conducted with the Second Professional students who had other subjects with summative assessments. The open-ended feedback from students on low participation in FC revealed that they prioritized reading the subjects that had upcoming assessments. Due to time constraints, some students were not motivated or interested in watching videos on FC of CMFM. A similar observation was made in a meta-analysis: students do not want to work at home and consider the pre-class videos to be burdensome in terms of time [7]. Some students could not open the MCQ link with the timer due to weak internet. 

How can the FC experience be improved?

Most students wanted FC in the Third Professional, where CMFM is one of the main subjects. Summative assessment of CMFM happens in the third year, so students have ample time to devote to the subject. Students wanted "double attendance" as a reward/incentive for attending FC. Most students have electronic gadgets, such as notebooks, so they wanted that, in addition to video, the text should also be provided as resource material for making annotations. A few students suggested an assessment along with FC for more participation.

Perception of the teacher

The teacher observed that FC can be effective if all students comply. Otherwise, the students will remain confused during the class. All students should be on the same platform during active participation in teamwork. FC can be effective for easier topics. Statistics is a comparatively difficult topic; some students did not understand it. FC can be effective in small-group teaching. Motivation of students is a critical factor in knowledge gain in FC. A teacher needs lots of preparation and time to implement an FC. The above views of teachers resonate with the results of a study by Nichat et al. [17]. A review by Oudbier et al. concluded that an FC is unsuitable for learning difficult topics because the learner must be able to understand the basic concept by themselves before the class [18]. Flipping the classroom fosters richer educational experiences and the possibility of in-depth learning in some, but not in all learners [19]. Although the FC is popular in other streams, it may not be as conducive in medical education because of years-long preclinical, paraclinical and clinical integrated courses, varied hierarchy, and experience of instructors. FC in medical education will require engaged students, motivated faculty, and enabled positive institutional transformation with a change management approach [20,21]. Realizing the optimal potential of the FC will need change management strategies at the lecture, course, and curriculum levels. It is necessary to incorporate changes into medical education through research projects and disseminate the results of new teaching methods and competency-based educational outcomes [22].

Many studies and a systematic review and meta-analysis reported that students demonstrated positive attitudes toward the FC, and scores in knowledge and skills acquisition were found to be similar or better than DL methods [4,23-26]. An FC could be considered a component of medical education by utilizing situational factors for a conducive learning environment, sequencing content for appropriate cognitive load, and scaffolding practices [21].

## Conclusions

The attrition rate was primarily attributed to the voluntary participation in research by undergraduate medical students, prioritizing subjects with upcoming summative assessments. In the CMFM undergraduate medical students in this study, there was an inconclusive difference in score improvements between FC and DL. The FC model demands time. As assessment drives learning, students prioritize time in subjects with upcoming examinations.

Therefore, the FC model in CMFM must be planned during the Third Professional, in which students take the subject seriously as they have a summative assessment. FC would be appropriate for small groups teaching easier topics. Change management approach at the level of lecture, course, and curriculum of medical education would tap the benefit of FC.
